# Personalization: a new political arithmetic?

**DOI:** 10.1080/1600910X.2022.2098352

**Published:** 2023-01-04

**Authors:** Sophie Day, Celia Lury, Helen Ward

**Affiliations:** a Anthropology, Goldsmiths, University of London, London, UK; b Centre for Interdisciplinary Methodologies, University of Warwick, Warwick, UK; c School of Public Health, Imperial College London, London, UK

**Keywords:** Personalization, political arithmetic, participation, precision, prediction, continuous present, vague whole

## Abstract

Scholarship on the history of political arithmetic highlights its significance for classical liberalism, a political philosophy in which subjects perceive themselves as autonomous individuals in an abstract system called society. This society and its component individuals became intelligible and governable in a deluge of printed numbers, assisted by the development of statistics, the emergence of a common space of measurement, and the calculation of probabilities. Our proposal is that the categories, numbers, and norms of this political arithmetic have changed in a ubiquitous culture of personalization. Today’s political arithmetic, we suggest, produces a different kind of society, what Facebook CEO Mark Zuckerberg calls the ‘default social’. We address this new social as a ‘vague whole’ and propose that it is characterized by a continuous present, the contemporary form of simultaneity or way of being together that Benedict Anderson argued is fundamental to any kind of imagined community. Like the society imagined in the earlier arithmetic, this vague whole is an abstraction that obscures forms of stratification and discrimination.

Now the Observations or Positions expressed by Number, Weight, and Measure, upon which I bottom the ensuing Discourses, are either true, or not apparently false, and which if they are not already true, certain, and evident, yet may be made so by the Sovereign Power, *Nam id certum est quod certum reddi potest*, and if they are false, not so false as to destroy the Argument they are brought for; but at worst are sufficient as Suppositions to shew the way to that Knowledge I aim at.

William Petty, *Political Arithmetick*

## Introduction

Scholarship on the history of statistics provides us with an understanding of the crucial role of political arithmetic (William Petty 1899 [1690]) in the emergence of classical liberalism, a political philosophy in which subjects are perceived as autonomous individuals with separate interests in an abstract system called society. This society and its component individuals became comprehensible and governable through the growth of statistics, the emergence of a common space of measurement (Desrosières 1998) and an avalanche of printed numbers (Hacking 1982; Porter 1995). Probabilities enabled commensuration, and classifications of normal and abnormal emerged through a comparison of population-level distributions (Hacking 1991). Our proposal, on the basis of a four-year study of personalization in the UK in and across digital culture, health care, and data science, ^1^ is that the categories, numbers and norms of this statistical political arithmetic have changed.

This new political arithmetic of personalization involves novel modes of counting, classifying and ordering, changing the ways in which publics are mapped onto populations (Cohen 2016; Hart 2010), and reconfiguring relations between the economy, politics and culture. In doing so, it produces a different kind of society, what Facebook CEO Mark Zuckerberg calls the ‘default social’,^2^ or what Nelms et al. describe as ‘a vision of the social without society’ (2018, 25).^3^ We address this new social as a ‘vague whole’ (Guyer 2014a) characterized by a continuous present and new forms of wealth creation such as the class of assets we call personalized generics. The continuous present is a contemporary form of the simultaneity or way of being together that is, so Benedict Anderson (2006 [1983]) argues, fundamental to any kind of imagined community.^4^

The term political arithmetic goes back to Sir William Petty’s volume, largely written in 1672, published in 1690, titled: *Political Arithmetick: Or, a discourse concerning the extent and value of lands, people and buildings*.^5^ However, as Bernhard Rieder notes, the use of arithmetic as a means to address size, complexity and uncertainty has a much longer history, including Luca Pacioli’s (fifteenth century) *Summa de arithmetica, geometria, proportioni et proportionalità*, which ‘standardized and disseminated double-entry book-keeping while popular *algorism*i, manuals or learning arithmetic, proposed practical methods that were both enabling and responding to the needs of increasingly complex forms of trade, such as dealing with logistics and planning, with diverse units and currencies, and with the distribution of risks and profits’. Rieder continues, ‘The requirements of long-distance trade, the emergence of larger commercial entities, and a general rise in organizational complexity elevated computation to the status of an empirical science’ (2020, 164).

Petty himself was a key figure in the English colonization of Ireland and one of the founders of the Royal Society. While he advocated the use of numbers for the purposes of government in contradistinction to a reliance on ‘superlative words’ and ‘intellectual arguments’, historians such as Mary Poovey (1993) have argued that the systematic application of arithmetical reasoning – listing, tabulating, and calculating – to decision-making in government and commerce was aided by specific rhetorical techniques. In what follows, we aim to describe the characteristics of personalization that demonstrate both continuities and differences with earlier forms of political arithmetic. These characteristics relate to recent transformations in the collection and analysis of data, the introduction of dynamic feedback loops in diverse, iterative, and automatic information and data processing systems including distributed ledgers and platforms of a variety of kinds, with different kinds of memory systems, from biobanks to administrative data sets and records. Such memory systems multiply relations of equivalence and difference or similitude and dissimilitude and expand the scope of comparison as a social relation through an explosion of metrics and scales. They deploy an understanding of change as not only constant and heterogeneous, but as an epistemic resource for action and decision-making.

The contrast we draw between old and new is necessarily over-stated, and we recognize that political arithmetic has never been a fixed body of knowledge or techniques but has developed in a variety of ways at different times in different places. Jane Guyer, for example, helpfully emphasizes that elements of platforms are ‘found’ as well as ‘made’ and carry legacies and logics that may not fit together readily. ^6^ As she and many others have noted, statistical and other calculative techniques have developed significantly over the last two hundred years in conjunction with the emergence of new modes of observation of publics and populations. However, we use the term ‘new political arithmetic’ to draw attention to what we consider to be an inter-related set of changes in relations between individuals and society that are of considerable significance.

In this new political arithmetic, we will argue, government is no longer organized as if subjects were indivisible, autonomous individuals, but in relation to fractal persons, that is, entities with relationships integrally implied. Such relationships are recursively scaled in quantitative and qualitative measures of similarity across a variety of on- and off-line platforms. This fractal person, we propose, is increasingly the subject and object of business and government, market and state, with relationships not simply implied or assumed but practically implicated or folded into digital and other environments, monetized in a variety of ways, and made available for research, monitoring and surveillance. In this new political arithmetic, society is not the additive sum of autonomous individuals; instead, individuals and societies are jointly distributed, multiplied and divided in practices of personalization across diverse and competing scales. Our proposal then is that the new political arithmetic does not rely upon (the ideology of) the indivisible subject (alone), but rather (also) addresses an always already divided subject, who can be scaled in a variety of self-similar ways to produce many ‘alls’.

While nobody was ever ‘just’ an autonomous individual, attempts were made to represent the subjects of government as such in the political arithmetic of classical liberalism. Recognizing the problems posed for policy by the fiction of autonomous individuals, Guyer identifies what she calls the household person, or the gross domestic person:

So the consumer of the CPI and GDP is not an individual in some philosophically autonomous sense, but a ‘household person’. This ‘household persons’’ long-term commitments to other members are assumed, but have stayed off the radar screen of formal data-gathering, since they are not assumed to be directly monetized. (2014b, 18)

Addressing the current situation, Guyer is led to ask, ‘Does the empirical rise of an intermittent isomorphism between individual and household create a momentary resolution between the household-person working practices of GDP technologies, the individualism of decision-theory ideology, and new dynamics in the transactional world, as long as one keeps the operative temporal frames short?’ (2014b, 18–19). Our suggestion is that this kind of resolution certainly exists but that the ‘long term commitments’ of persons are now much more visible on radar screens, making them available not only for monetization but also for forms of government. That is, persons are now routinely addressed as dividuals – distributed in relations by fractal logics across multiple scales – as well as continuing to be accorded the status of individuals. As the examples discussed below will indicate, this doubled address is sometimes invisible or unproblematic, but sometimes it is hard to achieve, leading to contesting claims to identity, new forms of politics, inequality and wealth creation.

## Personalization

Personalization is pervasive in everyday life in the UK and elsewhere: we are invited to participate in personalized medical, health and care services, receive personalized customer experiences, and find our way with maps that are continuously updated with information about our movements, our likes and dislikes. We are individuated in the rankings of Airbnb and Uber, participate in personalized learning, and travel on trains and planes at personalized prices (Moor and Lury 2018). We post selfies, share personal data in networks with friends and strangers, and create multiple personae in social media. At the same time, the rights long accorded to corporations as legal persons are now being extended to natural entities such as rivers, mountains and forests (Eckstein et al. 2019; Stone 1972). Currently, legal debates are exploring the extension of free speech to digital entities performing what are described as communicative acts.^7^ Yet while there is a growing body of literature on personalization in specific domains (for example, in health, Dickenson 2013; Prainsack 2017; in digital culture, Cohen 2016; Kant 2020; and in education, Williamson 2017), there is little analysis of how it operates *across* domains and its uneven significance for how we live together under old and new forms of inequality.

In earlier work (Lury and Day 2019), we described algorithmic recommendation – which we understood to be a paradigmatic technique of personalization – as involving a new mode of collective individuation (Simondon 1992), specifically, a dynamic pathway of classification and reclassification of ‘People Like You’. We emphasized that the classifications of ‘People Like You’ are simultaneously one and many, singular and plural, individual and collective; a personalized group, category, or generic emerging from the mapping of publics onto populations and vice versa (Cohen 2016). In our current research, we have built on this picture of a process of classification, de- and re-classification, and identified its reliance on three P(ractice)s. These Ps, we suggest, feed into each other, combining and recombining to make personalized types or genres of ‘People Like You’. The 3Ps are: a mode of address that involves inviting and formatting Participation*;* the inter-relation of liking (preference) and likeness (similarity) to produce Precise categories or classifications; and the testing and re-testing of these categories for the purposes of Prediction. We further suggest that this dynamic process of classification acts as a distributive logic with a moving ratio^8^: sorting people and things, informing processes and allocating resources along a variety of scales. For example, for Precision to enable Prediction, categories are classified and reclassified, and grouped and regrouped in an iterative process of Participation. It is this moving ratio – in which individuals and societies are jointly and continuously multiplied and divided – that we describe as the new political arithmetic.

However, we also observe that combining Participation, Precision and Prediction happens in all sorts of ways. As the variety of histories of personalization in the UK and elsewhere demonstrate there are many different actors involved, each with their own, sometimes very different, purposes. There are also very many examples of failed personalization and of partial, incomplete personalization.^9^ In addition, the priority given to different Ps in combination varies. For example, the eligibility criteria for the study of cancer in EBLIS that we describe below create Precision only insofar as they set the parameters for any subsequent Predictive value. Nevertheless we think the description of personalization as a political arithmetic is helpful because it identifies common practices across domains and allows us to explore differences within as well as between domains. It gives us analytic purchase on issues such as whether and how personalization contributes to old and new forms of stratification and discrimination, the possibilities it affords for new forms of wealth, changing relations between public and private, and the transformations in conceptions of personhood that it brings about.

The Ps we identify – Participation, Precision and Prediction – are widely recognized to be important to personalization – for example, P4 medicine is described by Hood and Friend (2011) as Predictive, Preventative, Personalized and Participatory. But our proposal is that it is the combination, and specifically, the sequencing of the Ps that is important for personalization as a political arithmetic. We emphasize the operation of Ps one *by* means of another and consider personalization in terms of P *by* P *by* P. In short, rather than seeing personalization as the outcome of simply adding Ps, our suggestion is that *how* and *in what order* they operate each other is what matters.^10^ Our study suggests that this ‘by the by’ logic operates in terms of measures of similarity, specifically those made possible by the measurement of ‘liking’ and ‘likeness’, that is, preference and resemblance. The fractal scaling of measures of similarity – rather than the aggregation of units of sameness that characterized the old political arithmetic – creates a variety of moving ratios in which persons are continuously and recursively distributed with different but mutually constitutive implications for the futures of stratified groups of People Like You. In the new political arithmetic of personalization, individuals cannot simply be summed as a society as in the old political arithmetic; instead, societies and individuals are mutually dividuated in a fractal and distributive logic to create personalized kinds, types or generics of a variety of sizes and shapes.

## P *by* P *by* P

To explore the operation of this logic we start with Participation, since most people encounter personalization through an invitation to Participate. An address to ‘you’ from entities or the use of our (first) name from an entity we do not know implies the phrase ‘People Like You’. These are speech acts which invite – seduce, cajole, demand, make it hard to refuse – a response (Chun 2016; Kant 2020). Collecting examples from a variety of domains, we have found significant variation in the style, scope and media of address associated with these invitations to Participate which lead to – or perhaps more accurately are designed to enable – a variety of data collection practices.

**Figure 1.**
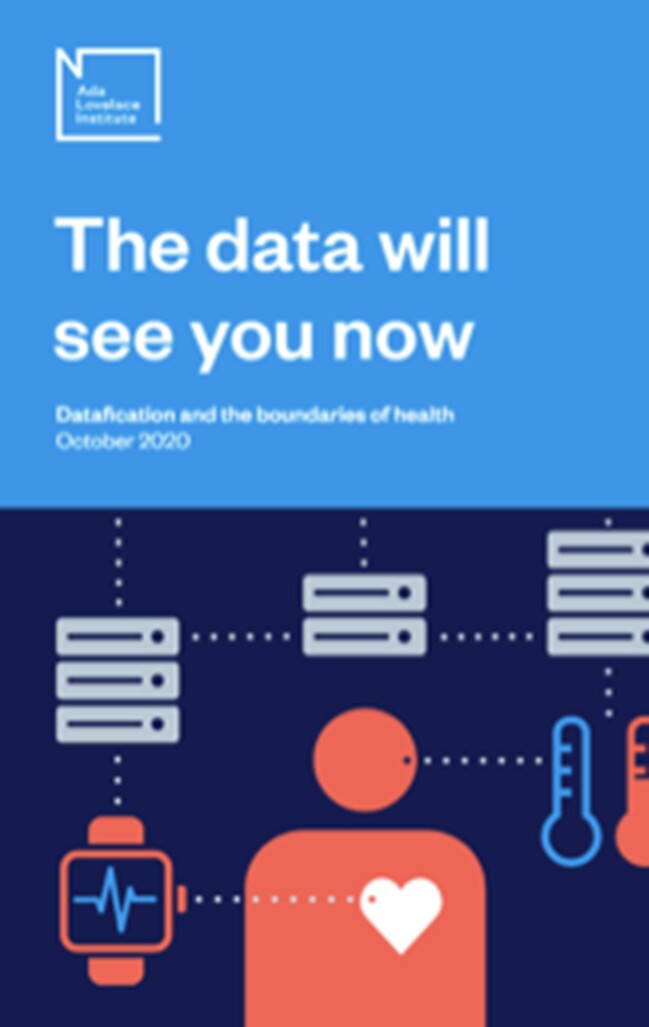
Cover of report produced by The Ada Lovelace Institute, 2020 (available at: https://www.adalovelaceinstitute.org/report/the-data-will-see-you-now/).

**Figure 2.**
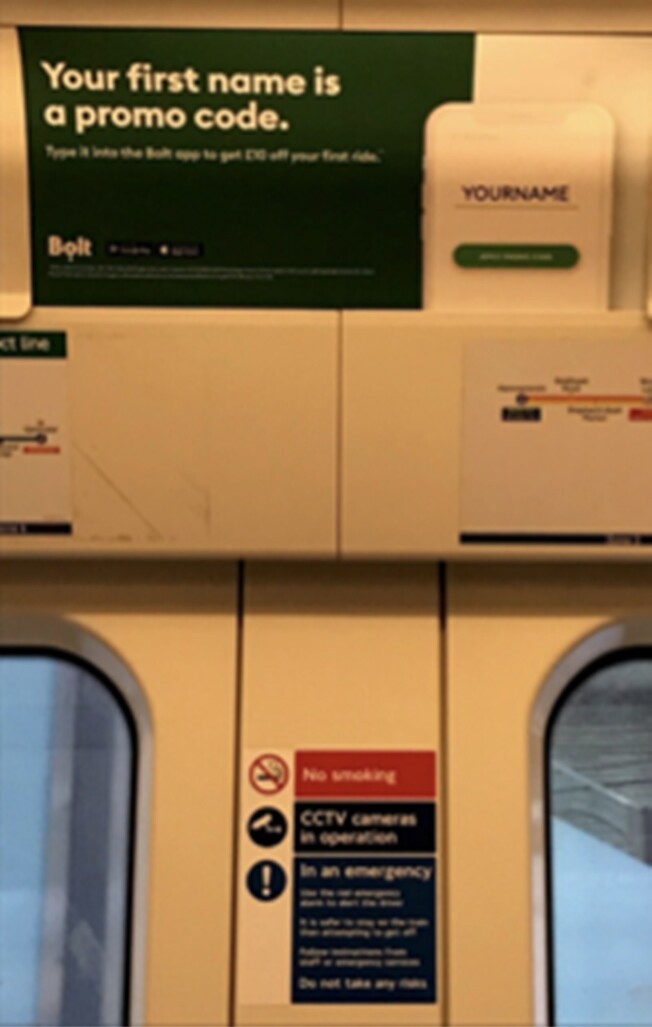
Advertisement. Photo credit: Celia Lury.

The mode of address formats Participation (Figures 1 and 2); it inscribes or frames the relevance of the invited Participation in relation to a function or purpose*:* for example, to segment a market, to proclaim solidarity with others, or to define criteria for a new therapy based on results of a clinical trial. In this regard, the invitations to Participate that are characteristic of the political arithmetic of personalization are part of a long-term shift from practices of classification that rely on understandings of essence or substance to those assuming the constitutive effect of relations of difference, and the operation of such relations in terms of functions of a variety of kinds (Cassirer 2003 [1910], Totaro and Ninno 2014). This long-term process has recently been accelerated by developments in computation; indeed, Rieder (2020, 17) considers computational techniques to operate in the ‘medium of function’.

The formatting of Participation may or may not enact a power to enforce compliance, but always involves ‘terms and conditions’ that circumscribe which actors can do what, as well as determining access, privacy, confidentiality, ownership of and licence to use the resulting data. In varied and diverse forms of address, Participation can be active and passive, knowing and unknowing. Any activity – such as on- and off-line shopping, campaigning, checking the weather forecast, interacting with friends, participating in a clinical trial – can be registered as Participation. Not responding to an address or invitation may still be counted as Participation if it is registered and logged in the iterative processes of data collection.

**Figure 3.**
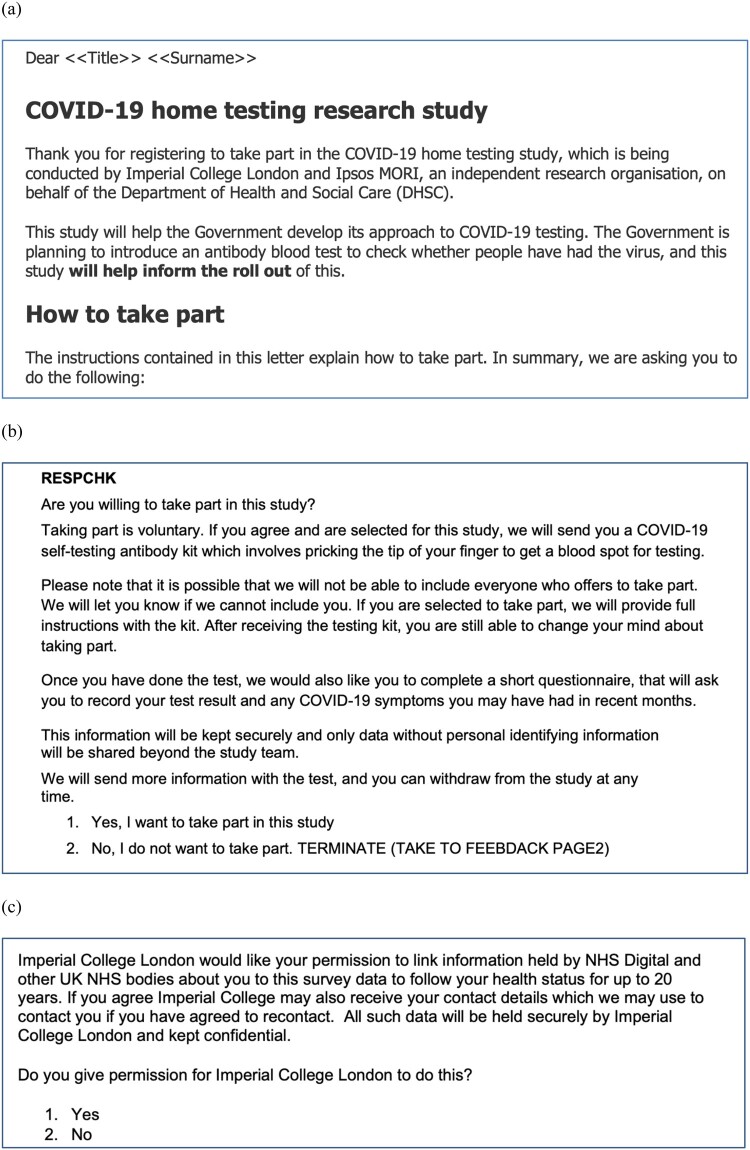
Examples of (a) invitation letter (b) consent to participate, and (c) consent to data linkage from the UK REACT (Real-time Assessment of Community Transmission) study; available online.

In the UK REACT (Real-time Assessment of Community Transmission) COVID study of 2020–2022, one of our case studies, randomly selected individuals were invited to participate (Figure 3), and around 30% agreed to participate in REACT-2, a study of antibody prevalence based on self-testing. As part of the study analysis, response rates were calculated by age, sex, geographic area and area-level deprivation. Using NHS data on all individuals registered with a family doctor, participants were compared to those who did not respond. Low participation by some age groups led to experimental approaches to see if a more targeted form of address (such as different wording in the invitation letter according to age) would alter the likelihood of response. Thus, even when ignoring the invitation, people were still Participating since data were collected and used in an iterative way to shape future invitations to Participate.

**Figure 4.**
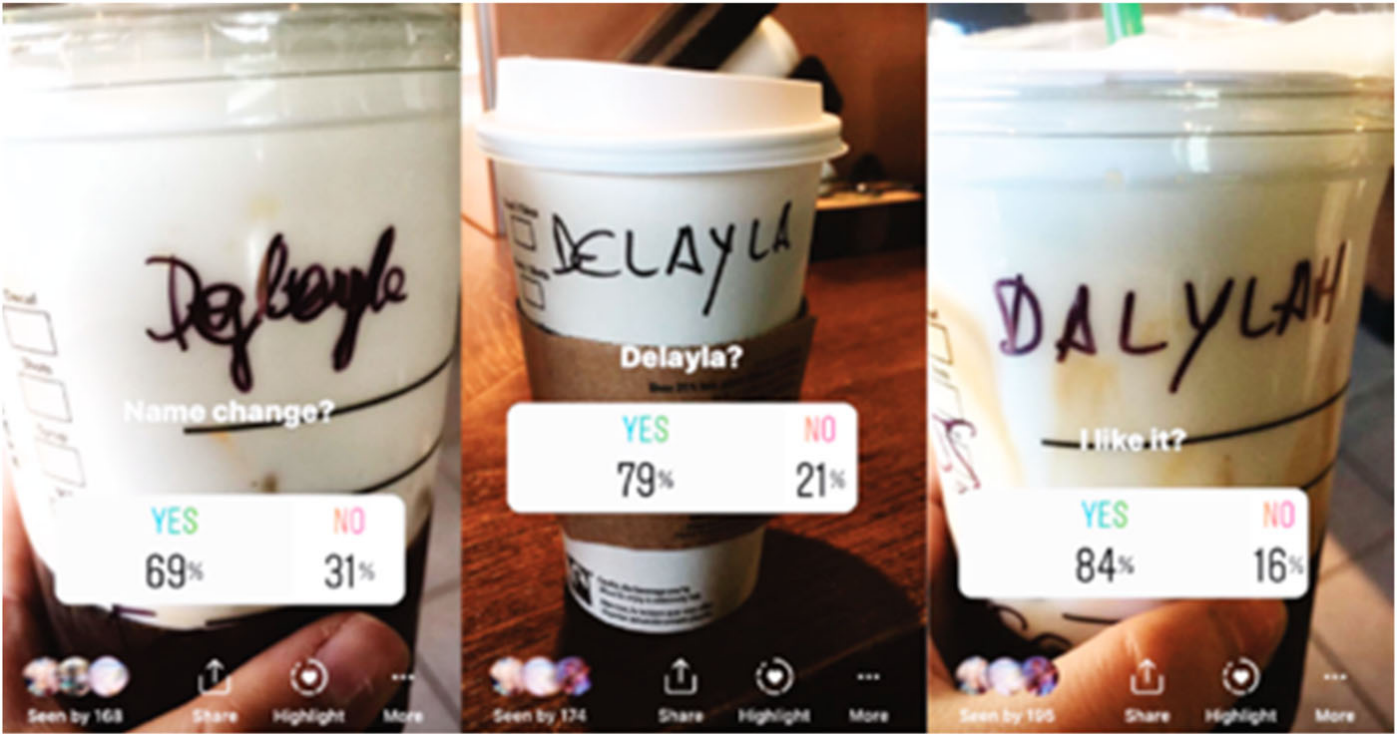
Photographs of ‘personalized’ coffee drinks. Photo credit: Delilah Niel.

Receiving invitations to Participate, we are invited to feel that we are being addressed as unique individuals. However, while the mode of address may respond to and feed into what Dominique Cardon (2019) describes as ‘an expressive demand for singularization’, the address is typically dependent on – and then in turn feeds into – a group or category of some kind. This can happen in a variety of ways: the group or category can be small or large, of interest to some, to many or only a few, as in the case illustrated below (Figure 4), in which the approximately 200 followers of one individual are grouped into those who like or dislike proposed changes in the spelling of her name.

**Figure 5.**
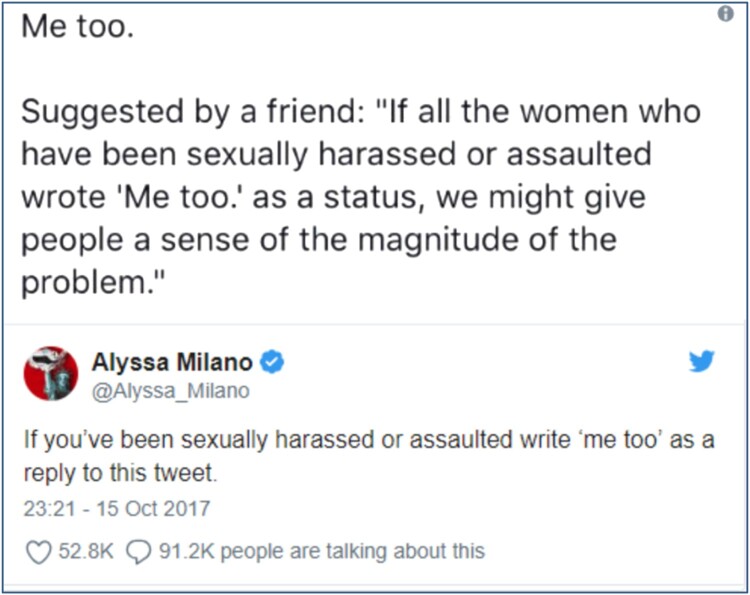
Tweets about ‘me too’.

The resulting segments or categories may never be named but are sometimes described by reference to established socio-demographic categories or recognized as genetic types, machine or digital phenotypes in medical and psychological research (M’charek 2020; Eshaghi et al. 2021). They also emerge in a less directed way as dynamic collectives or social movements as in the case of #longcovid or #JesuisCharlie and #MeToo (Figure 5) (Lury 2021; Lury forthcoming). The figure of speech or person that is so constituted is not composed as a collective entity of unique, independent ‘ones’, but as a moving ratio of more-*and*-less-than, simultaneously singular and plural self-similar persons. This is a different kind of count to that identified in the old political arithmetic.

**Figure 6.**
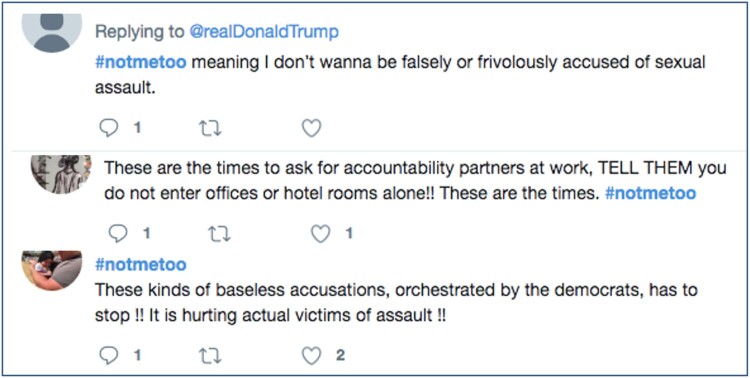
Tweets using ‘#not me too’.

Often, Participation is designed to be ongoing to provide data that may be folded back into the initial stratification, once again according to function or purpose. For example, health programmes, drawing on methods from marketing, develop and use increasingly Precise categories, as in targeted HIV prevention interventions (Gomez et al. 2019). In addition, in the new political arithmetic of personalization, segments, groups or strata are not as stable as previously, and can be – although they are not always – infinitely adjusted to whatever task is at hand, speeding up and multiplying the possibilities for representation and intervention associated with the old political arithmetic. In the REACT study, mentioned above, data linkage to results from repeated tests, subsequent infection or hospitalization is being used to refine the precision of the relevant categories. Data collected for one purpose can be and is increasingly repurposed by a variety of actors (Figure 6).

## Liking and likeness

Across all the domains we studied, we found that personalization emerges from the ways in which Participants, their responses and activities are tracked and registered, and then classified in relation to a fractal logic of liking and likeness. ‘People *like* you *like* things *like* this’ is the generic phrase we use to capture the fractal (that is, self-similar rather than self-same) basis of the making of these categories and associations. Importantly, our investigation shows that ‘liking’ and ‘likeness’ emerge from a diversity of practices and are inter-related in many different ways.^11^ Liking or preference can be as various as liking, tagging or following in social media, time spent on a specific webpage or advert, responses to A/B website testing, adherence to or adverse reactions to medical, pharmaceutical or other interventions. Likeness or similarity is also measured in well-established and newly emergent ways, through a variety of statistical and other calculative techniques, including testing to identify measures of proximity which inscribe membership in categories (Seaver 2021; Chun 2021).^12^ In consequence, preferences and similarities are differentially registered across domains and, in some settings, categories are derived from preferences while in others, they are registered as similarities.

Of course, it has long been recognized that liking and likeness overlap and that the one indicates the other (Bourdieu 1987). What is new is the extent and ease with which their association can be tested and one inferred or imputed from the other. In diverse practices, the grounds for establishing categories are being extended enormously, and recursively, since categories derived from each or both of liking and likeness are calibrated in sequence and combination to produce many kinds of People Like You. Constantly changing categories can now also be tracked more easily over time and across datasets, proliferating the ways in which inclusion, exclusion and belonging in these categories are operated by the collection and aggregation of data. Indeed, while we started this account of personalization with a focus on Participation for the sake of simplicity, we have already entered the matrix of P by P by P, which can itself be extended with other P(ractice)s: for example, Participation is frequently guided by a concern with the Precision of the categories produced in the inter-relating of liking and likeness, with the intent of both Predicting and Prescribing some future action or behaviour.

Whole fields of practice are being transformed in this way even though the recursive operation of P by P by P can – and often does – lead to double b(l)inds and dead-ends (Day and Lury 2017). What is sometimes called Precision medicine, for example, is associated with the new taxonomies and aetiologies that are emerging in molecular analysis, which, it is hoped, will improve Prediction using earlier classifications based only on symptoms and signs (Katsnelson 2013). Today, an anatomically defined breast cancer may be considered more similar to a prostate or ovarian than to another breast cancer although the accuracy of these new, more granular (molecular) cancer categories remains uncertain.

Test-retest processes have aligned some newer and older markers for a given category of cancer, led to the substitution of molecular for previous biomarkers, and facilitated the development and implementation of some new evidence-based treatment pathways. However, cancers often evolve in response to treatments because sub-dominant cancer clones are unaffected and become dominant in turn. So even if a particular cancer is initially sufficiently Precisely identified to Predict what treatment will be effective based on molecular analysis, this classification is provisional because treatment will change a cancer’s characteristics. Treatments in turn change periodically in response to emerging evidence, which is hard to collect even though most cancer patients now Participate in one way or another in research (Day et al. 2021). In general, however, ‘master protocols’ (such as umbrella and basket trials) and adaptive trial design now often *pre-emptively* address a heterogeneity that previously would have been defined post-facto through data gathered during the trial (FDA 2018, 2019). New trial designs inform decisions about which arm of a trial is to be opened or closed and who is to join which part, according to emerging findings as well as risks for individual patients.^13^

Not surprisingly, many medical studies and trials involving P by P by P are designed to search for actionable moments, as with an observational study that we followed, EBLIS (Exploratory Breast Lead Interval study). This study sought to understand better how to prevent relapse with metastatic disease. Women at high risk of relapse were followed over time to detect fragments of tumour DNA in their blood against their own specific gene panel, each woman acting as their own control. Preliminary results showed that it is possible to establish a ‘molecular’ relapse before a clinical diagnosis could be made (Coombes et al. 2019). This gap, up to two years in some cases, constitutes the ‘lead interval’ that may enable ‘actionability’, that is, earlier or pre-emptive treatment. To establish the Predictive value of this personalized method for detecting relapse accurately, however, the study team have to correlate their measurements with those used in usual care and establish the superiority of their own measures by following some Participants to what currently counts as relapse. A clinical trial of early as compared to standard interventions will ultimately show the value of this particular Precision by Prediction method, as personalization by Participation inches towards reliable actionability. More Precise diagnostic categories are intended to inform – Predict – optimal management; that is, results from the continuous tracking of outcomes are used to adjust those categories. So Precision emerges from as well as enables Prediction in the complex sequencing of this new political arithmetic of personalization.

The desire for Prediction by Precision is what motivates many instances of personalization inside and outside medicine (Mackenzie 2017). It is both cause and consequence of innovation in methods that not only change practices of classification but also *how* variance or distributions of attributes or features within a population are established (Amoore 2013; Hopman and M’charek 2020). Indeed, while Cardon (2019) suggests that statistical categorization has been called into crisis by the individualization of social processes, we have found that they continue to be at least to some degree inter-dependent: for example, some techniques of inter-relating liking and likeness benefit from the increasing interoperability of data updated in practices of Participation that are individualized in a variety of ways.

As noted above, AI and machine learning significantly extend the number and range of techniques to create and recreate groups, proximities and neighbourhoods of various kinds^14^ which are then further tested in different genres of Participation. Across all the domains we studied Participants are enrolled (knowingly or unknowingly) in a never-ending process of fine-tuning, extending and calibrating results. The resulting categories may be ephemeral: Cardon (2019) notes that events or traces of digital Participation are compared without categorizing them at all in real-time digital advertising auctions: ‘Instead of stable, durable and structuring variables that fixed statistical objects within categories, digital algorithms prefer to capture events (a click, a purchase, an interaction, etc.) that are recorded on the fly to compare them to other events, without having to perform a categorization’.^15^ In a research hospital, this may be a much slower process but some health systems are also nearing real-time with, for example, daily data updates as patients Participate in care, creating automated data processing across different platforms so that analyses can be applied rapidly through the generation of alerts indicating possible sepsis, for example (Honeyford et al. 2020).

However, while we found that a variety of old and new techniques co-exist, we suggest that there is something novel in the political arithmetic of P by P by P. While there was always a process of updating and pausing or stabilizing the categories to facilitate testing and retesting of associations, the process that Hacking (1986) describes as looping is now both speeded up and multiplied. There is, as Cori Hayden puts it in a study of the pharmaceutical market in Mexico, ‘a confounding and generative categorical abundance’ (forthcoming: 21). As we have shown, classification may involve the repetition of an address or invitation in relation to a short- or long-term purpose in practices of personalization, and the making of associations can be more or less open, interrupted or suspended. But, if it continues, it creates an emergent pathway of (different kinds of) similarities or resemblances among ‘People (more or less likely to be) Like You’.

Consider, for example, the emergence and acceptance of new types of ovarian cancer in a London university hospital by way of a discussion about research at a Science Café in November 2018. A clinical researcher in ovarian cancer and a researcher in mass spectrometry were discussing new analytic techniques with an audience at a cancer non-governmental organization. The clinician commented on the mass spectrometry presentation, ‘Yes, well, … I’m a simple oncologist but you are saying that cancer is infinitely complicated. Are there not exit points from this complexity where we can have some impact?’ He continued, and we summarize his comments:

After the early breakthroughs in ovarian, we found simply complexity in genomics. Everything was different from everything else. … The whole point of these big data algorithms is that they can work on multiple dimensions and so reduce the complexity to produce patterned data, that is, readable data. These techniques are multidimensional. Using, as does Amazon, non-negative matrix factorisation, (we) found seven patterns driven by seven mechanisms in ovarian cancer.

Further discussion revealed the importance for both researchers and audience of shifting between a Precise view that was ‘close up’ and produced a fine-grained, granular magnification that rendered the cancer unique as well as Precisely rendered, and a more distant view that aimed for an accurate approximation of the relations between one cancer and others to inform decisions about treatment.

But more than the simple multiplication of categories, the new political arithmetic of personalization involves a kind of looping or feedback in which the co-existence of multiple temporalities becomes more significant. Indeed, our suggestion is that in the new political arithmetic of personalization, it has become more possible to experimentally map multiple temporalities – of anticipation, obviation, and pre-emption – onto multiple categories in such a way as to maximize avenues for understanding, intervention and monetization. The calculated coming into co-existence of many temporalities in P by P by P is what allows for many mutually constitutive groupings to be simultaneously present at any moment in time, providing new ways to close the gap between correlation and causation, and offering new opportunities for actionability.

Of course, the significance of temporal reasoning for understanding relations of cause and effect is not in itself new. It is, for example, an important feature of Bayesian statistics (named after Thomas Bayes who formulated a specific case of Bayes’ theorem in a paper published in 1793). As described by Cardon, in this way of thinking, ‘what is defined *a priori* must be revisable by observing subsequent events. Bayesian reasoning allows the variability of the explanation of actions by tracing back from the effects to an assessment of the degree of certainty of their causes’. But Cardon also points out that ‘This variability can [now] be achieved by making models more flexible and by using heterogeneous data sets to compare ranges of correlations between data that vary according to context’. He gives the example of products offered by the automobile insurance industry that adjust ‘the premium to the number of kilometres driven (“pay as you drive”)’ (Cardon 2019). In the case of EBLIS discussed above and many others we observed, the prior distribution is re-calculated as more data becomes available, and this prior distribution is rolled into the next posterior, enabling the relations established through testing and retesting to be distributed to suit certain outcomes. Predictive utility is being retrofitted to the relevant input (Amoore 2020).

Adrian Mackenzie observes that in today’s data multiples there is a ‘layering, convolution and multiplication of relations that generates new dynamics’ (2010, 8). He uses the term distributive numbering to describe some of the novel algorithmic techniques that have these effects, including MCMC (Markov Chain Monte Carlo, a development of Bayesian statistics) which, as he notes, is increasingly used to identify ‘the operational or control points in … many practical settings (asthma studies, multiplayer game coordination, epidemiological modelling, spam filtering, and so on)’ (2016, 131). One distinctive aspect of such techniques, Mackenzie argues, is the combination of epistemic and aleatory understandings of probability. Significantly, MCMC and other novel algorithmic techniques are also more able to deploy the sequentially consequential aspects of Bayesian approaches because the new digital infrastructures of Participation (Kelty 2020) associated with platforms make more data more easily available more frequently. However, as we discuss below, while some of the methods may be new, the purposes, profits and ends of this re-distribution are familiar since the historical legacies and logics of the old political arithmetic continue to affect the relevance or purpose of the new.^16^

As we have noted, how the equation of ‘liking’ and ‘likeness’ is achieved by such techniques varies hugely according to the calculative space in which it is produced. In all cases, however, the value of the calculations is only realized as they are tested repeatedly in relation to data collected via techniques linked to many different kinds of Participatory methods and metrics. And with the rise of machine learning methods and AI, particularly deep learning, not only can possible posterior distributions be multiplied and tested (and re-tested) in relation to constantly changing priors but distributions can also be put into multiple relations with each other with the consequence that ‘People Like You’ are distributed and redistributed across model compartments in diverse categories and clusters. Mackenzie concludes, ‘If individuals were once collected, grouped, ranked, and trained in populations characterized by disparate attributes (life expectancies, socio-economic variables, educational development, and so on), today we might say that they are distributed across populations of different kinds that intersect through them. Individuals become more like populations or crowds’ (2016, 118).

Cardon suggests that what he calls hypothetical-deductive machines are being replaced, with the rise of AI, by inductive machines. Rather than direct replacement, we suggest that relations between theory, deduction and induction are currently the source of experimentation. Indeed, we consider that contemporary developments in methodology are engaging with a new version of the ‘problem of induction’ that Mary Poovey (1998) describes in her account of the emergence of the modern fact, aiming to identify the ‘suppositions’ that William Petty proposed should be ‘not so false as to destroy the Argument they are brought for’. For example, in another of our case studies, both classical statistics and machine learning approaches were applied in Predictive models for long COVID, based on analysis of the population of Participants and of symptoms. Logistic regression was used to identify associations between various individual factors such as sociodemographic characteristics and the reporting of persistent symptoms at 12 weeks, and gradient boosted tree models were used to investigate how adding variables altered Predictive ability. Additional machine learning methods were then used to identify clusters of Participants based on symptom profiles at 12 weeks (Whitaker et al. 2022). These clusters were attached to Participants in the form of additional, new categories, which were then used to identify new associations. The clusters were found retrospectively to be Predictive insofar as these new, post-hoc categories were associated with initial severity of COVID-19 infection. As part of this still ongoing study, these new groups are now being tested further, and refined, and may be used to stratify people for further invitations to Participate and provide new data.

## Mapping populations onto publics

The recursive looping of populations onto publics in which categories of ‘People Like You’ emerge is, we found, hugely significant for the possibilities of identification, recognition and solidarity among those who are categorized. Take, for example, the creation of categories such as Facebook’s multicultural or ethnic affinities: ‘African American’, ‘U.S.-Hispanic’, and ‘Asian American’ (Phan and Wark 2021). From 2016 to 2020, these categories made use of data relating to Participation on the platform, including data relating to the preferences or likes expressed by users, to classify them as having an affinity for African American, U.S. Hispanic, or Asian American culture.^17^ Facebook explained:

The word “affinity” can generally be defined as a relationship like a marriage, as a natural liking, and as a similarity of characteristics. We are using the term “Multicultural Affinity” to describe the quality of people who are interested in and likely to respond well to multicultural content. What we are referring to in these affinity groups is not their genetic makeup, but their affinity to the cultures they are interested in.

The Facebook multicultural targeting solution is based on affinity, not ethnicity. This provides advertisers with an opportunity to serve highly relevant ad content to affinity-based audiences. (Quoted in Phan and Wark 2021) ^18^

Despite this statement, Facebook have been forced to drop these categories because their use reinforced racial discrimination. But their short-term existence is revealing. It points to a political arithmetic that creates attributes constituted in fractal relations of liking and likeness rather than socio-demographic characteristics (for example, gender, age and ethnicity). Nonetheless, their value arises in part because they can be – and are – continually superimposed on the latter, so that in this case race operates as an absent presence (Hopman and M’charek 2020).^19^

**Figure 7.**
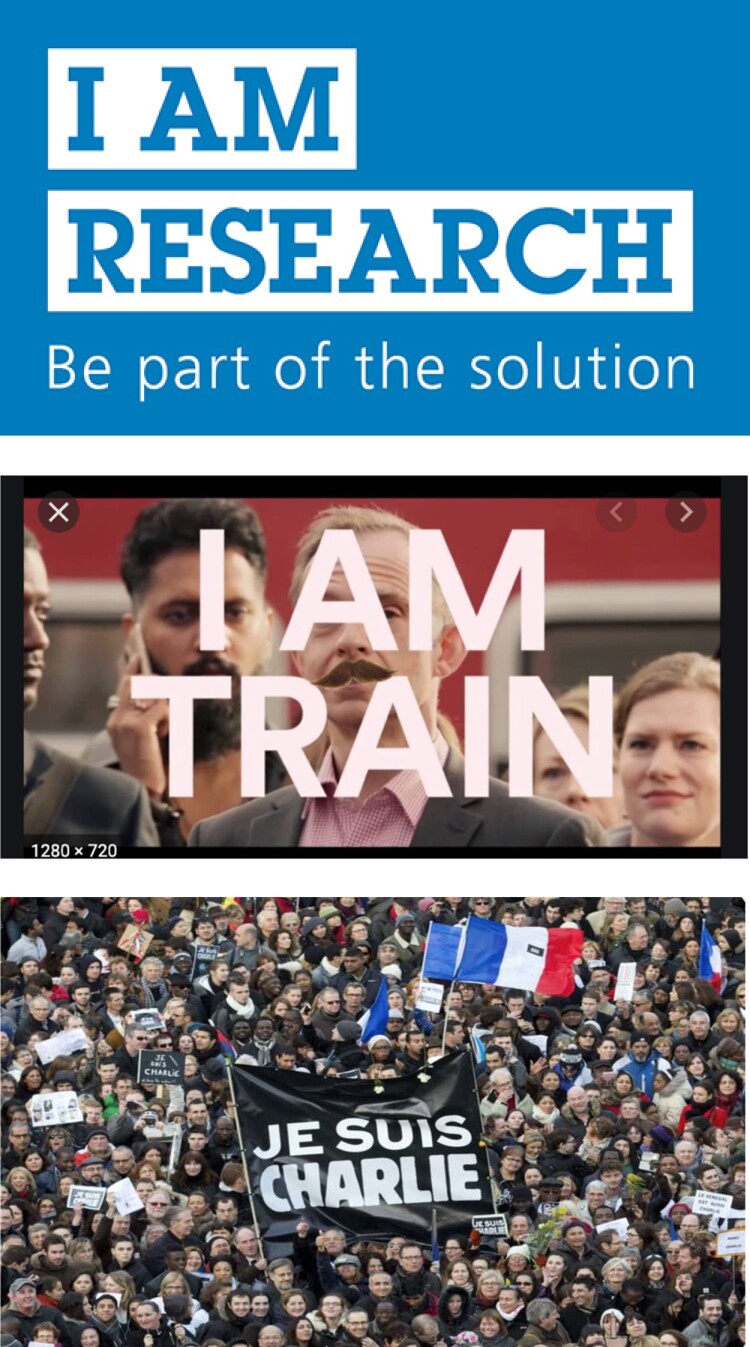
Examples of ‘I am’ campaigns. (a) NIHR I am Research campaign: https://www.spcr.nihr.ac.uk/news/nihr2019s-i-am-research-and-nhs70-campaign-kicks-off; (b) Trainline I am train advertisement: https://www.campaignlive.co.uk/article/trainline-i-am-train-anomaly-london/1363512; (c) *Je Suis Charlie* supporters: https://twitter.com/gilbertovaladez/status/1479541663005609994?s=12.

In other examples, the categories, genres or types produced in these mappings of publics onto populations are highly various – as in the examples (Figure 7) of ‘I am research’, ‘I am train’, #JesuisCharlie or ‘type of cancer responding to this type of treatment at this point in time’ – but all have a variety of implications for how people are included (or not), how they attach to or (dis)identify with a category or feel that they belong. This is not so surprising. As Cardon (2019) observes, classification is a primary mechanism by which a society is rendered intelligible to itself: it ‘provides representations, points of comparison and explanations that nurture the actors’ common sense’. But such common-sense understanding is now hard to establish because the new categories do not necessarily map onto established socio-demographic and other characteristics and they are often ephemeral.^20^

In a study in collaboration with colleagues in Chile that made experimental use of a prototype recommendation app, we found that participants employed not only what we might call established representational understandings of identity (‘[The app] was saying that it is not probable that I like Latin music and I like Latin music, I mean I'm Latin [laughs]’) but also more operational or ‘protocological’ (Cohen 2016) kinds of understanding:

There are other exceptions like Rdio [an extinct music platform], which was more precise [in comparison to Spotify], but I'm thinking that it's because I probably interacted more, as I'm the one who's constantly educating it. There's a kind of computer–human interaction that tells you, “Is this OK?”, “No”, “Ah, let's go on to the next one, is this OK?”, “Yes”, “Check” and it's fed by something that I'm doing explicitly.

For some of the participants, this fed into a more general understanding of personal identity as staged and contextual:

Because there’s the whole debate about, okay, algorithms, the way they take information about you, do they take too much, but there’s a whole other problem which is more like the problem of what is identity and, and I think what is good with [the] app is that it pushes you to think about, okay, how do I stage myself and this just shows that okay, even though there’s a sort of fantasy or authenticity and being true to yourself, actually, we spend most of our social life in staging our self in different ways. And also, there’s even like a sort of internalisation of being true to some rules of social roles. All those things are super naturalised. So, I think something good with recommendation is like, yeah, it makes you think about how you could appear.

In the case of the long COVID study we described above, the category of Long Covid traces its origins to the activities of those affected, who chose to self-identify: ‘Long Covid has a strong claim to be considered the first illness to be collectively made by patients finding one another through Twitter and other social media’ (Callard and Perego 2021), including the use of the hashtag #longcovid. In research co-produced with medical researchers, people identifying with Long Covid facilitated collection and analysis of data from which more Precise clusters have been identified using hierarchical clustering (Ziauddeen et al. 2021). At the same time, these Participants have ensured that the overall category is still live so they can continue to share experiences. This has meant that new Precise clusters can continue to be created in relation to the continually updated experiences of those identifying with Long Covid (Perego et al. 2020).

In medicine – unlike recommendation systems in retail – the accuracy of these more Precise categories is important; being included in a category of people who might like to buy something you do not like may be annoying (or funny or creepy) but it is rarely a matter of life or death. In the more informal discussion that followed the Science Café presentations mentioned above, people agreed that while they wanted to be ‘interesting’ and therefore invited to join a research study they did not want to be ‘unique’, in a Precise class of just one, since there would likely be no treatment available. ‘You don’t want to be first either … .’, ‘All these things, they have some toxicity that may not have been shown in testing before. The early stage of drug testing is a worrisome time’. Similarly, those affected with Long Covid often express a desire for an effective universal treatment while recognizing that more Precise sub-classifications or clusters may be the route to more effective targeted therapy (Ward et al. 2021).

For Participants, Precision medicine connotes a targeting that is the opposite of a one-size-fits-all treatment such as cytotoxic chemotherapy. But the accuracy of Precision targeting is uncertain and unsurprisingly ‘worrisome’ to Participants in the clinic until it has proven replicable: for example, until a targeted drug has been tested for long enough to make Prediction reliable and show that those with a particular category of cancer live longer and better when they take that drug rather than another. In other words, our study suggests that whether or how the accuracy of Precise interventions enables the identification of a functional invariance sufficient to support the making of a Prediction is what is at stake in contemporary personalized medicine.

## Prediction in/as the continuous present

While Prediction is widely seen as one of the most important – if not *the* most important – goal driving the use of statistics and AI in many fields (Mackenzie 2017; Cardon 2019), we found that what is meant by Prediction is changing insofar as the techniques described above fold relations to many possible futures into many possible priors (or pasts) to create what we call a continuous present.^21^ That is, while the practice of Prediction in the new political arithmetic of personalization co-exists with earlier understandings in which the present is stabilized and serves as a ground for Prediction of a certain future, it is also contributing to shifts in finance, medicine, policing and insurance as well as other domains (Esposito 2011) in ways that expand the possibilities of pre-emption (Amoore 2013). In the continuous present, many possible and more or less probable futures, previously unidentified, are available for intervention before they happen and are effectively disposed of, while at the same time other futures are continually anticipated and acted on through a test-retest logic.^22^

We understand this continuous present to be a contemporary form of the simultaneity that Benedict Anderson (2006) proposed is fundamental to any imagined community. He contrasts the ‘simultaneity-along-time’ of the pre-modern, in which promises prefigured a future to be fulfilled, with the modern time of the imagined community of the nation state. This modern time is a ‘transverse, cross-time, marked not by prefiguring and fulfilment, but by temporal coincidence, and measured by clock and calendar’ (2006: 24). As he says, ‘This new synchronic novelty could arise historically only when substantial groups of people were in a position to think of themselves as living lives parallel to those of other substantial groups of people – if never meeting, yet certainly proceeding along the same trajectory’ (2006: 192).

For Anderson, the use of ‘meanwhile’ recognizes the simultaneity of the imagined community of the nation state. This imagined community included some – those who read newspapers, used national transport networks or were conscripted into national service – and excluded others. Today, inclusion, exclusion and belonging in imagined communities of ‘People Like You’ – people with a certain breast cancer, with Long Covid, participating in #MeToo – happens differently. Our proposal is that ‘anyway’ takes the place of ‘meanwhile’ in the continuous present of personalization’s distributive logic. ‘Anyway’ effects an abrupt full stop, a swerve, another perspective on something and/or a change of topic. ‘Anyway’ orchestrates the (re-)calibration of the moving ratio of P by P by P, creating pivotal moments at which relations of inclusion, exclusion and belonging in an imagined community are distributed again and again in a ‘time [that] has gone from being represented as a lineal past-present-future continuum to being seen as punctuated and fragmented, oscillating between “fantasy futurism” and “enforced presentism”’(Guyer 2007, 410).

The imagined community here is no longer the nation, but rather, as Guyer (2014a) suggests, a vague whole. She uses this term to point to the indeterminacy and uncertainty of contemporary belonging. In the new political arithmetic, Participants are continuously enumerated Precisely to Predict multiple futures in ‘real time’, that is, ‘now’, but this happens in relation to a constantly changing and vaguely defined referent or community. In this enumeration, the territory or ground of belonging is constantly shifting. The punctuated calendrics (Guyer 2007) of test-retest and platform-based coordination of P by P by P amplifies ‘some voices and presences over others’, attracts ‘close collective access and attention’, enables ‘specific owners and engineers to reorient it for new purposes, and constitutes ‘a place for announcing originality’ (Guyer 2016, 4).

‘Anyway’ also marks a kind of speed up or updating, It creates a pause in order to proceed, ‘Anyway (let’s move on).’ What counts as ‘superfast’, ‘now’ or ‘real time’ in specific social and technical arrangements is inter-related with optimisation or actionability in a ‘right time’ (Riles 2004), that is, right for certain purposes in relation to particular interests. Teeming temporalities - from fintech to household cycles – are built from and into varied everydays.^23^ The sequencing of the real and the right thickens the ‘now’ of ‘anyway’ with a grammar of tenses to constitute the phantasmatic simultaneity of a continuous present. The vagueness of these imagined communities is not a deficit but a ‘provocation to action’ (Guyer 2007) in their constitutive incompleteness.

## Personalized generics

In our analysis so far, we have focused on how the possibilities for action provoked by the new political arithmetic of personalization are informed by shifting distributions. In this final section, we describe the forms of wealth with which it is associated and point to the importance of a class of assets that we call personalized generics. Assets are usually defined as tangible or intangible resources which are ‘controlled by the entity as a result of past events and from which future economic benefits are expected to flow to the entity’ (Burton and Jermakowicz (2015, 39) in Birch and Muniesa 2020, 3). In the P by P by P of personalization, however, assets are not simply ‘whatever can be sustained in value over time’ (Guyer 2016, 248), but also what can be sustained in value *in* time, that is, in the operation of time made possible by the mapping of populations onto publics in a continuous present.

As is widely recognized (Birch and Muniesa 2020), the multiplication of data sets together with the possibilities of both more regular and more rapid updating, and the operation of an increasingly wide variety of memory systems including distributed ledgers have led to new possibilities of wealth creation. The response to COVID-19, for example, has seen an acceleration of the assetisation of health data in the UK with the creation of vast resources that link research and routine data in Trusted Research Environments (see NHS Digital 2021; Faulkner-Gurstein and Wyatt 2021).^24^ One such case, Opensafely, claims to have achieved what the NHS failed to deliver over several decades by using,

… a new model for enhanced security and timely access to data: we don’t transport large volumes of potentially disclosive pseudonymised patient data outside of the secure environments managed by the electronic health record software companies; instead, trusted analysts can run large scale computation across near real-time pseudonymised patient records inside the data centres and secure cloud environments of the electronic health records software companies.^25^

While we recognize that this form of assetisation is linked to economization or neoliberalism, we found that the social, economic and political implications of the assets emerging from the distributive logic of P by P by P are highly varied. Some are branded but by no means all; they include, for example: #MeToo; #longcovid; digital and biological phenotypes including organoids and the ‘liquid’ biopsies collected in the EBLIS study discussed above; AMSR boyfriend role-play videos such as DennisASMR); Microsoft’s MyAnalytics; a ‘suspect population’ (Hopman and M’charek 2020); and #BlackLivesMatter.

**Figure 8.**
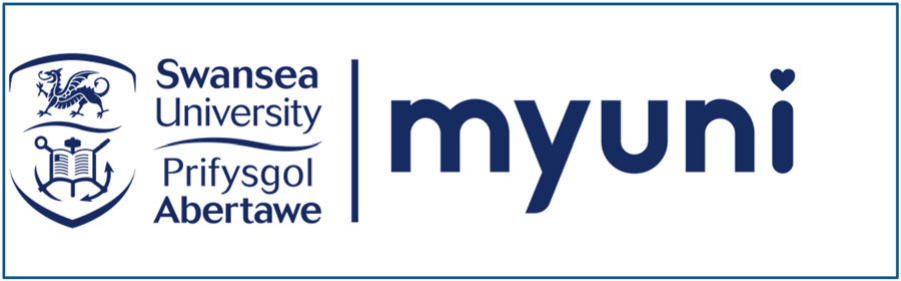
Swansea University MyUni logo: https://myuni.swansea.ac.uk/myunihub/.

Birch and Muniesa emphasize the importance of ‘the conditions [that assetisation] engenders’ (2020, 4), and we note the diversity of ways in which ‘different kinds of sameness’ (Hayden 2013) are being created in the inter-relation of liking and likeness associated with the political arithmetic we have described. Indeed, calling up Mary Poovey’s study of the *Genres of the Credit Economy* (2008), we use the term personalized generics to refer to such assets because of their potential to act as potent sites of ‘distinction, enchantment and stratification’ (Hayden 2022, 9; Hayden 2013; Lury forthcoming) as well as wealth creation. The term captures the ways in which persons may be genericized as in the making of categories of ‘People Like You’ and how generic collectives – such as the university – may be personalized through a formatting of Participation that relies on the use of personal pronouns, as in the case of MyUniversity (see Figure 8; Lury forthcoming).

**Figure 9.**
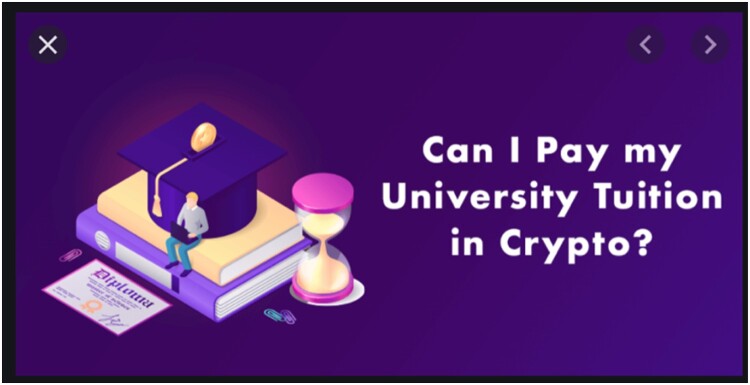
Advert for crypto https://www.coinmarketcal.com/en/news/can-i-pay-my-university-tuition-in-crypto.

To specify personalized generics as a distinct form of asset draws attention to the novelty of the ways in which the distribution – the administration, management and allocation – of resources occurs in the mapping of populations onto publics and vice versa. It recognizes the variety of such resources, including for example the narrative and other reputational resources that are so central to the making of individual, organizational and collective identity.^26^ It highlights the opportunities and difficulties involved in establishing protection for – and claims to ownership of – specific features or attributes of ‘People Like You’. That these (personal) attributes or features are not those currently protected in law – race, gender, disability and age for example – is producing a whole series of ongoing controversies, of which the case of Facebook’s multicultural affinities discussed above is only one example. But to recognize the assetisation of personalized generics also acknowledges what Cori Hayden calls the ‘almost infrastructural market force’ (2022, 10) that is required to assert ownership, including the creation of complex architectures of on- and off-line relations between companies, governments, platforms, apps and different types of participants (see Figure 9). These inevitably afford diverse relations between not only ‘publics’ and ‘populations’, but also ‘publics’ and ‘privates’.

## Conclusion

In the preceding sections we have sought to show how personalization relies on and is constituted in a distributive logic that emerges in the sequencing of practices of P by P by P. Giving examples from across the domains of digital culture, healthcare and data science, we have proposed that personalization is central to a new political arithmetic, providing the basis of novel forms of decision-making, bureaucracy, governance and political collectivity as well as assetisation. While we have described personalization as a new political arithmetic, we have also tried to show that its logic has a history and that it is neither totalizing nor unitary. Rather, as we have indicated, it is the subject of ongoing political debate and dispute.^27^ While futures are anticipated and obviated in the continuous present that personalization calls into existence, alternative and multiple ecologies of recognition, identification and belonging co-exist as Participants are variously (un)able to play with, resist and rewrite the moving ratios of P by P by P. At the same time, recognizing the existence of these multiple ecologies, we also point to the fact that many of the instances of P by P by P we have studied are closed loops and walled gardens – gated or semi-gated – and produce multiple, overlapping inclusions and exclusions as well as different opportunities for belonging (Nelms et al. 2018).

**Figure 10.**
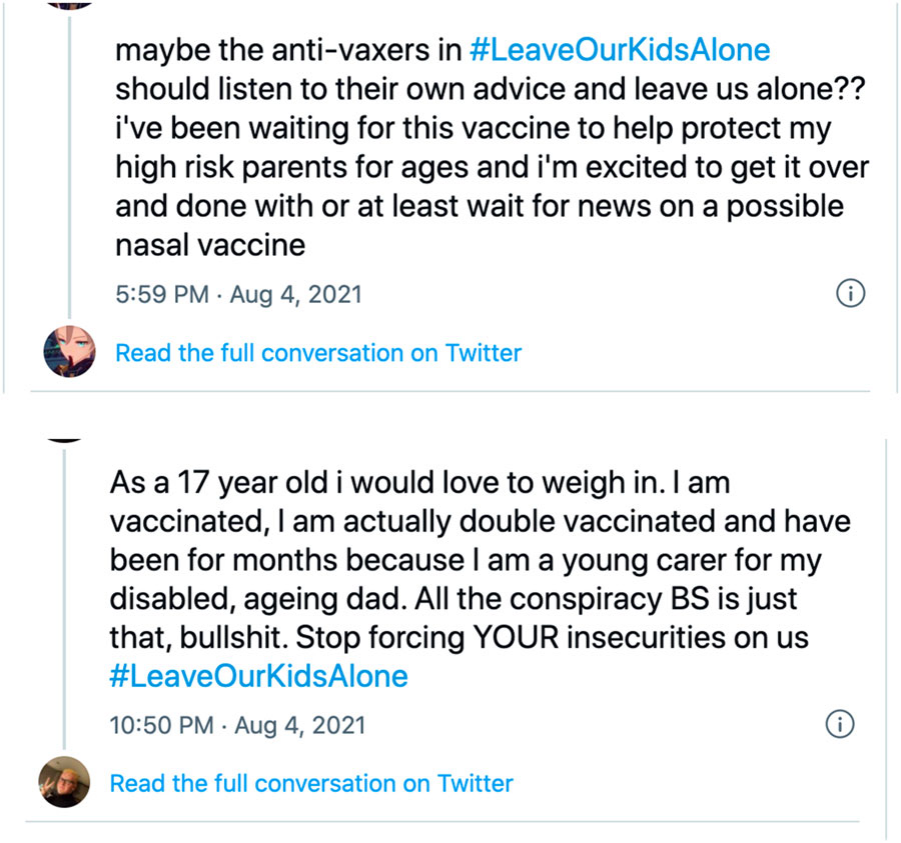
Tweets about vaccination.

At the heart of this politics is the transformation of our understandings of the person in the making of a default social. The new political arithmetic does not rely upon (the ideology of) the indivisible subject, but rather combines it with an always already divided subject, who can be scaled in multiple ways to produce many vague wholes or ‘alls’. Indeed, we argue that in the new political arithmetic of personalization it is a fractal person who is increasingly the unit of the loop for business and government, market and state. A variety of self-similar relations are not simply implied or assumed but *activated* in the moving ratio of this loop to constitute a default social. Whether, how, and by which actors such relationships are stabilized or institutionalized provides opportunities for intervention, action, solidarity and assetisation for some, while precluding or blocking it for others. Each of the vague wholes or ‘alls’ of People Like You is reciprocally if asymmetrically distributed in relation to a variety of differently positioned ‘ones’ so that while some ‘alls’ are ‘smaller’ or ‘larger’ than others, even the largest does not contain each and every ‘one’ (see Figure 10). At the same time this ‘one’ is also always potentially an(other) ‘all’: #BlackLivesMatter: #AllLivesMatter; #BelieveAllWomen: #NotAllMen.

As markets and states engage with global phenomena such as Covid, climate change and financialization, and populations are stratified and publics are scaled in new ways, the social, economic and political complexities of counting fractal persons become increasingly evident: in the new political arithmetic there is no easy way to reconcile ‘one for all’ and ‘all for one’.

## Data Availability

Institutional ethics approval was obtained for REACT-2 from South Central–Berkshire B Research Ethics Committee (REC ref: 20/SC/0206; IRAS 283805) and for Personalisation in Breast Cancer Medicine and Healthcare from North West - Greater Manchester West Research Ethics Committee, (REC ref: 18/NW/0550; IRAS 248517).

## Notes

1 In this paper, we present a range of examples from this study (see http://www.peoplelikeyou.ac.uk) to illustrate our understanding of personalisation as a new political arithmetic. While the aim is to indicate some of its scale and scope, we do not mean to imply personalisation is a unified phenomenon.

2 In 2010, Zuckerberg explained how Facebook’s ‘Open Graph’ made the web intrinsically relational through ‘like’ buttons and other plug-ins.

3 Nelms et al write, ‘This is a very different notion of the social from that imagined in 19th or 20^th^ century social theory and social action. The social of a ‘just us’ economy is not the statistical artifact of an aggregation of individuals in a national population, which gave rise in the 19th century to a host of efforts to measure and police public and private vices, from criminal behavior to unemployment, and which provided the conceptual underpinnings for the modern welfare state (Donzelot, 1979, 1984; Ewald, 1986; Foucault, 2003; Hacking, 1990). Quite the contrary, it is envisioned as the product of a disaggregation, a granular personalization of preferences and taste profiles that can be algorithmically sorted and targeted (Cheney-Lippold, 2011; Pasquale, 2015; Seaver, 2012; Tiessen, 2015)’ (2018: 25).

4 In drawing on Anderson’s notion of imagined community, we recognise the criticisms of his approach to the emergence of nations, including his use of nationalism to understand developments in colonised countries and his lack of acknowledgement of other kinds of imagined community (see, for example, Guha 1994; Hobsbawm 1990). As with any way of ‘being together’, inclusion or a sense of belonging always also involves exclusions. Anderson acknowledged many criticisms of the imagined national community in a later (2006) edition of the book.

5 The full subtitle of *Political Arithmetick* is: *Or a Discourse Concerning,* *The Extent and Value of Lands, People, Buildings: Husbandry, Manufacture, Commerce, Fishery, Artizans, Seamen, Soldiers; Publick Revenues, Interest, Taxes, Superlucration, Registries, Banks, Valuation of Men, Increasing of Seamen, of Militia's, Harbours, Situation, Shipping, Power at Sea, &c. As the same relates to every Country in general, but more particularly to the Territories of His Majesty of Great Britain, and his Neighbours of Holland, Zealand, and France*.

6 Guyer notes that a platform ‘is made up of built components and applications, from which actions are performed outward into a world that is not itself depicted’ (Guyer 2016, 114).

7 We emphasize this point to make it clear that while our focus is on the scaling of ‘people like you’, this classification is inter-related with the scaling of ‘things like this’, as in the reversible phrase ‘people like you like things like this’, which complicates everyday understandings of the social. An example here concerns transformations in the payments industry in which ‘No longer simply moving funds from one account to another, payment implicates the person and his or her digitally mediated social milieu’ (Nelms et al. 2018, 17; see also Hart 2005, 2017). As the authors note, the actors in this process invoke a new understanding of the social – ‘just us’, even as they seek ‘to disappear into the very relations they facilitate’ (2018: 15).

8 We use the term ‘moving ratio’ somewhat differently to Paul Rabinow in his discussion of the contemporary (2007).

9 See, for examples, Algoglitch, and note too the perverse results of sorting by body weight, fish and water consumption that led the West Virginia Manufacturers Association (2019) to say that West Virginians might not be representative of other Americans in relation to Environmental Protection Agency recommendations. The Association apparently argues that West Virginians can tolerate higher levels of toxic chemical pollution in a state long compromised by coal mining and chemicals (Fagan 2019). More recently in 2021, a ‘serious incident’ was reported on a Tui airflight when those titled Miss were classified as children with a ‘standard weight’ of 35kg (*The Guardian* 9 April 2021).

10 The sequencing of the operations of addition, subtraction, multiplication and division in arithmetic is indicated by the use of brackets: this sequencing is significant for the summing of a whole that may then become the input for a further calculation.

11 The use of liking and likeness are instances of what Barbara Stafford (1999) identifies as the longstanding use of analogy as an associative method, a demonstrative and evidentiary practice.

12 They now include, for example, techniques of unsupervised machine learning for clustering, which lead to clusters based on unspecified similarities that can then be used to identify a ‘cluster medoid – the representative observations at the centre of each cluster’ (Hennig and Liao 2013). Clustering done repeatedly and automatically identifies ‘stable’ clusters even though there can be substantial within-cluster heterogeneity (ibid).

13 The Bayesian Continuous Individualised Risk Index predicts individual patient pathways and outcomes in breast cancer through integrating serial measurements of varied biomarkers over time (Kurtz et al. 2019).

14 As Cardon (2019) describes, the development of predictive methods based on granular behavioural, genomic and other ‘big’ data are currently being driven by the ‘renunciation by AI techniques of the idea of programming symbolic rules organizing reasoning and causality in favor of sub-symbolic methods of adaptive and oscillating computations.’

15 Cardon (2019) continues, ‘While the Internet user is loading the web page he wants to consult, his profile is auctioned by an automaton so that robots programmed by advertisers compete for the best price to place their banner ads. The operation lasts less than 100 milliseconds. The auctioned profile is not one of those typical portraits of traditional marketing. It is not based on variables such as age, occupation or income level. … Unlike the measurement of opinions or subjective expressions on social networks, computing infrastructures oriented towards personalized prediction are mainly concerned with traces of conduct: clicking, buying, reading, moving, etc.’

16 William Petty’s infamous recommendations of transmutation to make the Irish more (like the) English was initially fitted to a Protestant and subsequently to a Catholic English sovereign (McCormick 2007). The proposed transmutation was designed to travel first in one direction and then in another: first, it was imagined that Catholic women would become Protestant if they married English men; then, with a change in monarch, Protestant men would become Catholic if they married Irish women. In short, it was assumed that what is now often called homophily (Kurgan et al. 2019) - relations of affinity and similarity - required heterophily, which in this context involved differences in gender.

17 This example shows how affinity (calculated on the basis of Facebook’s likes) can become a proxy or marker of likeness, that is, an indirect criterion for deriving membership in a category, just as an affinity in Precision Medicine for a treatment enables likeness to be provisionally inferred among cancers.

18 See Geoghegan’s (2021) discussion of the ‘affinities’ between the cultural analytics of social media networks and the structural anthropology of the 1950s.

19 This is to follow Amade M’Charek’s (2020) understanding of race in terms of technologically mediated relations between individuals and the population, and her concern with the ways in which versions of race change as they travel across sites.

20 Cardon (2019) notes that the ‘process of individualization has … contributed to individuals' criticism of the taxonomies in which they are categorized.’ Whether this is to be seen in terms of an expansion of possibilities for self-definition or as multiplying the dimensions along which people can compare themselves and be compared is an ongoing question.

21 In UK English, use of the present continuous tense expands the ‘simple present’ and suggests a temporary situation that has a dynamic and/or repetitive momentum.

22 Esposito (2011) argues that the future is both an intersection and combination of the present future and the future present.

23 *The Financial Times* (Fletcher and Wigglesworth, April 20, 2021) reported that hedge funders have been unable to predict what will happen next during the pandemic. Computer driven hedge funds rely on analysts being able to understand how the different assets they trade will move in relation to each other. One industry insider reportedly said, ‘For global tactical asset allocation portfolios, your biggest friend or enemy is correlation’. Consider also the how truth is distributed across media platforms, courts of law and activists in the movement #MeToo (Lury 2021).

24 The related process of the branding of populations is developed by Aaro Tupasela (2017) in a discussion of the ways in which historico-cultural narratives of origin and authenticity have come to play an increasingly important role in medicine in the leveraging of populations as new types of scientific products. As he observes, ‘understanding populations as brands helps to identify the ways in which biobanks seek to leverage the sample collections they have onto international research markets’ (2016: 48).

25 See website, https://www.opensafely.org/

26 See Rosamond forthcoming, and her article in this Issue for an analysis of the reputational economy.

27 To give just one example, Carr and Lempert (2016) describe what they call ‘predatory scaling’, observing ‘how people’s attempts to be recognized as political or social groups are stymied as their collective claims are rescaled as assortments of individual ones’.
